# Protecting children from secondhand smoke: a mixed-methods feasibility study of a novel smoke-free home intervention

**DOI:** 10.1186/s40814-016-0094-7

**Published:** 2016-09-12

**Authors:** John Marsh, Ann McNeill, Sarah Lewis, Tim Coleman, Manpreet Bains, Alexandra Larwood, Jacqueline Purdy, Laura L Jones

**Affiliations:** 1 UK Centre for Tobacco and Alcohol Studies, Faculty of Medicine and Health Sciences, University of Nottingham, Nottingham City Hospital, Clinical Sciences Building, Nottingham, NG5 1PB UK; 2 UK Centre for Tobacco and Alcohol Studies, Institute of Psychiatry, King’s College London, Addictions Sciences Building, 4 Windsor Walk, Denmark Hill, London, SE5 8BB UK; 3 UK Centre for Tobacco and Alcohol Studies, Faculty of Medicine and Health Sciences, University of Nottingham, Medical School, Queen’s Medical Centre, Nottingham, NG7 2UH UK; 4 UK Centre for Tobacco and Alcohol Studies, Institute of Applied Health Research, University of Birmingham, Public Health Building, Edgbaston, Birmingham, B15 2TT UK

**Keywords:** Secondhand smoke, Smoke-free homes, Complex intervention, PM_2.5_, Feasibility study, Acceptability, Mixed-methods, Recruitment

## Abstract

**Background:**

Globally, 40 % of children under 14 years are regularly exposed to secondhand smoke (SHS), typically in their homes. There is limited evidence of the effectiveness of interventions to reduce children’s SHS exposure, and so the aim of this study was to test the feasibility and acceptability of a novel intervention to help parents and carers (caregivers) to reduce their children’s exposure to SHS at home.

**Methods:**

A novel multi-component intervention to support caregivers to reduce their children’s SHS exposure at home was tested in a two-phase feasibility study. The 12-week intensive intervention delivered in the home consisted of three components: behavioural support, nicotine replacement therapy (NRT) for temporary abstinence and feedback on levels of SHS exposure in the form of children’s salivary cotinine (phase 1) or home air quality (PM_2.5_) (phase 2). Participants were caregivers who smoked inside their homes and had at least one child under the age of 5 years living with them the majority of the time. Mixed-methods were used to explore the acceptability and feasibility of the intervention as well as processes, particularly around recruitment and retention, for an exploratory efficacy trial.

**Results:**

Twelve caregivers completed the study, all received personalised feedback on SHS exposure and behavioural support to help them to make their homes smoke-free and the majority at least tried NRT. Saliva cotinine results were variable in phase 1, and therefore, measures of PM_2.5_ were used for feedback in phase 2. Behavioural support was well received with personalised feedback reported as being the key motivator for initiating and maintaining behaviour change.

**Conclusions:**

Recruiting disadvantaged caregivers was labour intensive, but once recruited, this novel intervention was both feasible and acceptable in supporting caregivers to reduce their children’s exposure to SHS at home. It is appropriate to test the efficacy of this novel intervention in an exploratory randomised controlled trial.

**Trial registration:**

This is not applicable for the current study; however, a registered exploratory randomised controlled trial linked to this manuscript is currently ongoing (ISRCTN81701383).

**Electronic supplementary material:**

The online version of this article (doi:10.1186/s40814-016-0094-7) contains supplementary material, which is available to authorized users.

## Background

Globally, 40 % of children under 14 years are exposed to secondhand smoke (SHS), and of the 600,000 annual deaths linked to exposure, 28 % occur in children [1]. Children’s exposure to SHS takes place predominantly in the home [2, 3] and is largely attributed to parental smoking in the home [4, 5]. Whilst exposure in England has declined recently [5], 39 % of children who live with smokers are still regularly exposed to SHS at home [6]. Children’s exposure to SHS has been causally linked with a number of childhood morbidities [7–10], and it has been estimated to result in an additional 300,000 UK general practice consultations and 9500 hospital admissions annually [10].

The most effective way to reduce children’s SHS exposure is for their caregivers (parents and other carers) to quit smoking. However, when caregivers continue to smoke, the next best option is for them to make their homes smoke-free. A systematic review [11] of 57 interventions and a further meta-analysis [12] of 30 studies aimed at reducing children’s exposure to SHS found mixed evidence for effectiveness which was insufficient to recommend any one particular intervention strategy. There is a need for more high quality, robust studies with objective validation of children’s SHS exposure. Interventions based on behaviour change theories and involving intensive and sustained contact with smoking caregivers are reported to show the most promise [13]. In addition, a recent smoke-free homes (SFH) intervention study [14] has shown that providing personalised feedback on children’s SHS exposure as part of a motivational interview may have an effect on improving home air quality and thus reducing children’s exposure to SHS in the home.

Using the Medical Research Council (MRC) framework for complex interventions as a guide [15], we combined the findings from our previous research which explored ways to support families to initiate and maintain a SFH [16, 17] with current evidence on what may be effective components of SFH interventions, for example: intensive and sustained counselling constructed around sound behaviour change theory which focusses on changing participant attitudes and behaviours [13, 18–20] and developed a novel multi-component intervention to help reduce children’s exposure to SHS in the home. The intervention, which aims to support caregivers to reduce children’s exposure to SHS in the home, combines intensive behavioural support, personalised feedback on household SHS levels and the offer of nicotine replacement therapy (NRT), licensed for temporary abstinence, as a substitute for smoking in the home. The aim of the study was to assess the feasibility and acceptability of this novel intervention using a phased, iterative approach, to refine the individual components and their delivery in preparation for an exploratory controlled efficacy trial. The specific objectives of this study were to (a) assess participant recruitment and retention rates and (b) explore the acceptability and feasibility of the behavioural support, nicotine replacement therapy, and personalised feedback components of the intervention.

## Methods

### Phase 1 procedure

#### Design, setting and recruitment

A two-phase process was employed with refinement of intervention components as necessary between phases. Phase 1 ran from September 2011 to February 2012. Participants were recruited via Sure Start Children Centres (CCs) in Nottingham City, England, which are free to join and offer free or subsidised activities and support for low-income parents with children under 5 years. We chose to recruit via CCs as the smoking rates amongst caregivers accessing CCs are high [17]. At the time of the study, there were 16 CCs in Nottingham City; 11 of these gave us permission to recruit and we randomly selected five to recruit from. The five CCs which declined to participate in the research reported that this was due to competing priorities with service delivery or concerns that we would not see sufficient families who met the study inclusion criteria. The inclusion criteria required that participants smoked inside their home, were the primary caregiver of at least one child under the age of 5 years who lived with them, were not pregnant or breastfeeding, had a fixed address for the intervention duration, were not currently trying to quit smoking, were over the age of 18 years, had good spoken English and gave written informed consent to participate. At the end of the study, participants who had completed the whole study protocol were offered a £50 retail voucher to compensate for time given to participation. Other smoking adults living in the same house as the primary caregiver and able to be present at appointments were also invited to receive the intervention. Ethical permission was gained from the University of Nottingham Medical School Ethics Committee (ref: E/03/2011).

### Intervention (see Fig. 1)

Caregivers received a 12-week home-based intervention which aimed to support them to reduce their children’s exposure to SHS in the home and comprised of:

**Fig. 1:**
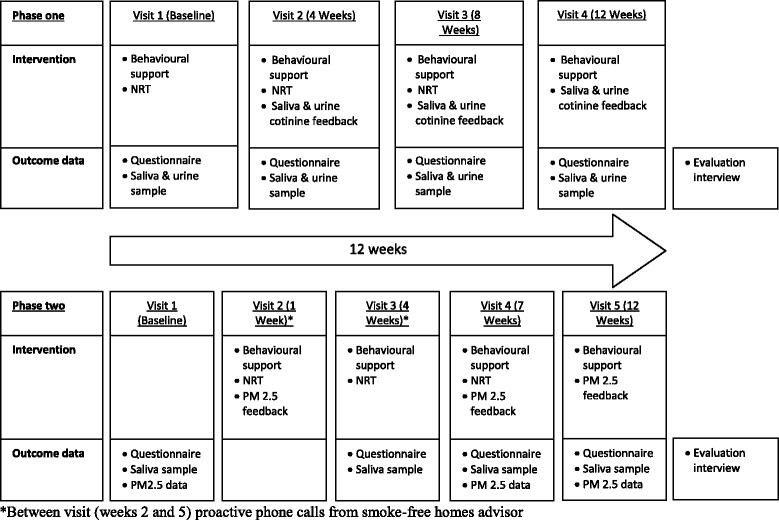
Diagrammatic representation of the two phases of the feasibility study

- Four sessions of face-to-face behavioural support (up to 60 min each) to promote changes to home smoking behaviours based on the principles of motivational interviewing, delivered in homes by a specialist SFH advisor. The support included caregiver education on the dangers of SHS, discussion of smoking behaviour and smoking rules in households, and identifying barriers to change and working with participants to elicit strategies to overcome barriers.
- Up to a 12-week supply of NRT to support temporary abstinence in the home (rather than cessation) by caregivers/other household adults. A variety of NRT products were offered, to permit matching to participants’ requirements, including use of NRT combinations. All were supplied at no cost to the participants.
- Urine and saliva samples were collected at each visit from an ‘index’ child (typically the youngest, but the primary caregiver was given the choice) and analysed for cotinine (a metabolite of nicotine, reflecting SHS exposure). Feedback on saliva cotinine was provided and explained at the subsequent visit (post-collection), and results were compared to levels expected from children with no SHS exposure (0–0.2 ng/ml), from children living with a smoker (6 ng/ml), and from the average level of a non-smoking bar worker heavily exposed to SHS (3 ng/ml) [21].

### Data collection methods and analysis

#### Quantitative data

##### Demographic questionnaires

Questionnaires were developed in-house and pilot tested with PPI representatives due to the lack of availability of validated questionnaires which covered each of the areas of interest within the evidence base. The questionnaires collected data on demographics, participant and child health and well-being, family and household home smoking behaviours, NRT use, and quit attempts, as well as beliefs around smoking in the home with children present. The questionnaires were administered at baseline and weeks 4, 8, and 12.

##### Cotinine measurement and feedback

Saliva samples from the index child were obtained using a Salimetrics Children’s Swab (Salimetrics Europe Ltd). The swabs were placed between the child’s inner cheek and gum until saturated. Urine samples were also collected from the index child in phase 1 by either a clean catch method (for children who were toilet trained) or via cotton pads in nappies. All samples were stored in a freezer at −20 °C before being transported at ambient temperature to ABS Laboratories (Cambridge, UK) for analysis of cotinine concentrations with a limit of detection of 0.1 ng/ml.

##### Quantitative analysis

Data from the interviewer-administered questionnaires were captured using SPSS version 14, and descriptive statistics were calculated. Raw cotinine data were inputted into Microsoft Excel, and descriptive statistics were calculated. Means and ranges were used to summarise continuous data that were normally distributed, and medians and ranges if data were skewed. Categorical data were summarised using counts and percentages.

#### Qualitative data

##### Evaluation interviews

Within 2 weeks of their final intervention visit, the participants were invited to take part in an evaluation interview with an independent researcher (MB). A semi-structured interview guide (see Additional file 1) explored participants’ views and experiences of taking part in the study, their general experiences of trying to create a SFH, views on each component of the intervention and how the intervention could be improved.

##### Qualitative analysis

Interviews were digitally audio-recorded and transcribed verbatim with each transcript subsequently checked to ensure data quality. Transcripts were analysed using thematic analysis [22]. AL and JP independently reviewed each transcript, and the initial ideas were noted that identified preliminary codes. These codes were then grouped into potentially relevant themes and discussed between the analysts, the interviewer (MB) and with the wider research team. Further analysis clarified the specific nature of each theme leading to the development of names and working descriptions (Table 1). Following the agreement of the identified themes, extracts were taken from the transcripts to illustrate each theme in order to reflect the experiences of the participants.

**Table 1:** Themes interpreted from the qualitative evaluation interview data

Core theme	Sub-themes
Acceptability and feasibility of behavioural support	- Approach and characteristics of the advisor
	- Face-to-face vs. telephone support
	- Sharing of knowledge
	- Practical support
	- Discussion of personalised feedback results
Acceptability and feasibility of nicotine replacement therapy	- NRT sample bags
	- Flexibility to change NRT
	- Advice on how to maximise effectiveness of NRT for temporary abstinence
	- Side effects
	- Using NRT to quit in future
Acceptability and feasibility of personalised feedback	- Urine vs. salvia cotinine
	- Sampling techniques
	- Air monitor set-up/data collection
	- Impact and interpretation of results
Length and structure of intervention	- Duration of the intervention and number of visits
	- Intervention intensity
	- Amount of information provided at each visit
	- Timing of visits within intervention period
	- Disengagement/withdrawal towards the end of the intervention
Facilitators and barriers to participation in the intervention	- Recruitment approach
	- Desire to quit
	- Impact of other adult smokers in the household and wider social network
	- Inconvenience allowance
	- Safeguarding of children

## Results phase 1

### Recruitment and retention

Recruitment for phase 1 was carried out over 14 weeks from 39 Sure Start CC sessions; 256 people were approached to assess eligibility, and 19 (7 %) met the inclusion criteria, of whom eight (42 %) were recruited. Only a small number of those caregivers who were approached to participate in the study were eligible as the majority reported being a non-smoker, and of those who reported smoking, only a small number reported smoking inside the house. Two participants were subsequently lost to follow-up (data not presented). Only one additional smoking adult who lived in a study household was recruited (data not presented). Semi-structured evaluation interviews were undertaken with three of the six participants. Caregivers who did not complete an evaluation interview were either not available to participate within the 2-week window at the end of the intervention or declined to take part.

### Study population

Participant baseline characteristics are presented in Table 2.

**Table 2:** Feasibility sample baseline demographic characteristics

	Phase 1 (*n* = 6)	Phase 2 (*n* = 6)	Evaluation interviewees (*n* = 7)
Age (years)			
Mean (range)	27 (23–31)	26 (21–34)	26 (21–34)
Ethnicity *n* (%):			
White British	5 (83.3)	4 (66.7)	4 (57.1)
Black or Black British	1 (16.7)	1 (16.7)	2 (28.6)
Caribbean			
Mixed-White Caribbean	0 (0)	1 (16.7)	1 (14.3 %)
Black Caribbean			
Age left full-time education (years)			
Median (range)	16 (14–25)	16^a^ (14–18)	16^b^ (14–19)
Highest level of qualification *n* (%):			
None	2 (33)	1 (17)	2 (29)
GCSE’s or similar	2 (33)	4 (67)	2 (29)
A/AS levels or similar	1 (17)	1 (17)	3 (43)
Degree or similar	1 (17)	0 (0)	0 (0)
Number of children in household			
Median (range)	2 (2–4)	1 (1–2)	2 (1–4)
Housing *n* (%):			
Rented	3 (50)	4 (67)	4 (57)
Local authority	3 (50)	2 (33)	3 (43)
Employment *n* (%):			
Homemaker/full-time parent	5 (83)	4 (67)	5 (71)
Full-time paid work	1 (17)	0 (0)	0 (0)
Full-time student	0 (0)	2 (33)	2 (29)
Cigarettes/day			
Median (range)	16 (8–30)	7 (3–20)	10 (3–30)

The totals may be greater than 100 due to rounding

^a^ *n* = 4 due to two participants still in full-time education

^b^ *n* = 5 due to two participants still in full-time education

#### Acceptability and feasibility of behavioural support

**Fig. 2:**
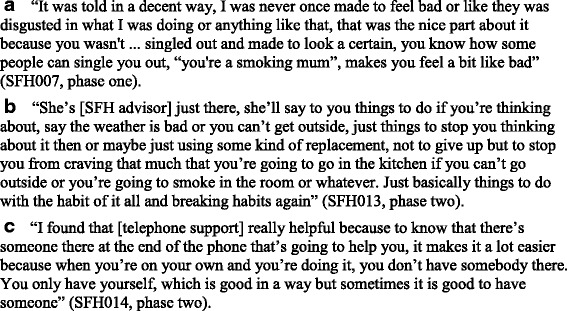
Acceptability and feasibility of behavioural support

Behavioural support discussions lasted between 20 and 60 min and were built around personalised feedback in the form of saliva cotinine. At 12 weeks, all caregivers self-reported that the behavioural support component was very (*n* = 2) or extremely (*n* = 4) helpful in supporting them to make their home smoke-free. All six participants ranked behavioural support at the second most effective and important component of the intervention package. In the evaluation interviews, the participants described the behavioural support they received from the SFH advisors as being important with a non-judgemental approach being valued; resulting in them not feeling stigmatised for smoking in their homes or pressured to change their behaviour (Fig. 2a). Instead, the participants recalled that the SFH advisor encouraged and supported them in creating a SFH. This support was particularly important in the early stages or when participants experienced difficulties maintaining changes for reasons such as bad weather which acted as a barrier to going outside to smoke (Fig. 2b).

#### Acceptability and feasibility of nicotine replacement therapy

**Fig. 3:**
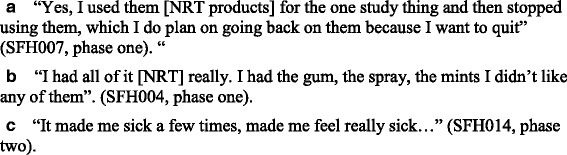
Acceptability and feasibility of nicotine replacement therapy

At baseline, all six caregivers stated that they planned to take up the offer of NRT to support them to make their home smoke-free. Between baseline and week 4, five participants reported that they had taken up the offer of NRT and reported using the product/s (all five were using the inhalator). Between weeks 4 and 8, five participants reported that they had taken up the offer of NRT and reported using the product/s (one was using an inhalator, one was using an inhalator and quick mist, one was using an inhalator and patches, two were using patches). Between weeks 8 and 12, four participants took up the offer of NRT of which three reported using the products (one was using an inhalator, one was using an inhalator and quick mist, one was using an inhalator and patches, and one was using patches). The participants reported predominantly using NRT to help with cravings for short period of time and for helping them to cut down without planning to quit smoking. As part of the interviews, some participants stated the NRT was useful and added that they planned to use the products to quit smoking in the future having had a positive experience using NRT for temporary abstinence (Fig. 3a). In contrast, other participants reported problems such as side effects resulting in them not using the products (Fig. 3b).

#### Acceptability and feasibility of personalised feedback

**Fig. 4:**
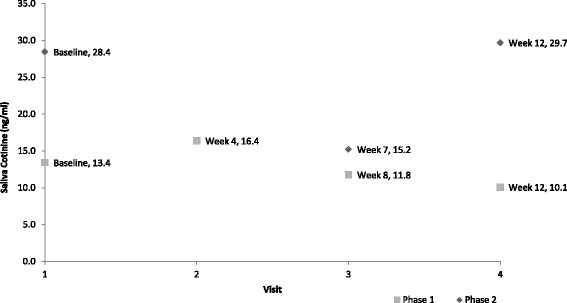
Average saliva cotinine results

**Fig. 5:**
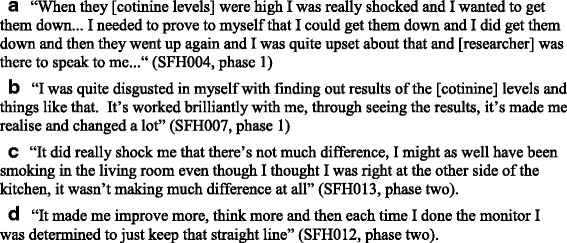
Acceptability and feasibility of personalised feedback

A total of seven (of a possible 24) urine samples were collected (data are not presented). The collection of urine samples was problematic with some children unable to provide a sufficient sample volume during the visit and this could be distressing for caregivers and their child. All 24 saliva samples were successfully collected and analysed. The average saliva cotinine for the six participants at each time point is shown in Fig. 4. On average, saliva 12 cotinine declined by 3.3 ng/ml (range −25.8 to 18.8 ng/ml) between baseline and week 12. The participants reported, as part of the interviews, that collecting saliva from children was acceptable and non-intrusive. However, although the collection of saliva for cotinine analysis from young children was acceptable, the results were variable, not consistent with corresponding urine cotinine results, and often did not reflect the home smoking behaviour changes self-reported by participants. Receiving feedback on saliva cotinine was emotive for a number of participants, with some reporting that high cotinine levels were distressing. However, such results appeared to prompt several participants to make changes to their home smoking behaviours (Fig. 5a, b).

### Learning from phase 1: protocol review

A review of the study design, processes and results was undertaken by the research team at the end of phase 1. The review led to the intervention being slightly modified for phase 2 to help further improve acceptability and feasibility and continue to inform the development of an exploratory efficacy trial. The length of the intervention remained 12 weeks; however, the timing of the visits throughout the 12 weeks was changed (see Fig. 1), increasing the frequency of the visits and helping to maximise support in the early weeks. To further support caregivers proactive telephone support from the SFH advisor was introduced in the second and fifth weeks. We introduced the opportunity to sample different types of NRT during the second visit to help ensure that participants were prescribed the most suitable product/s to support them to make their home smoke-free between visits.

**Fig. 6:**
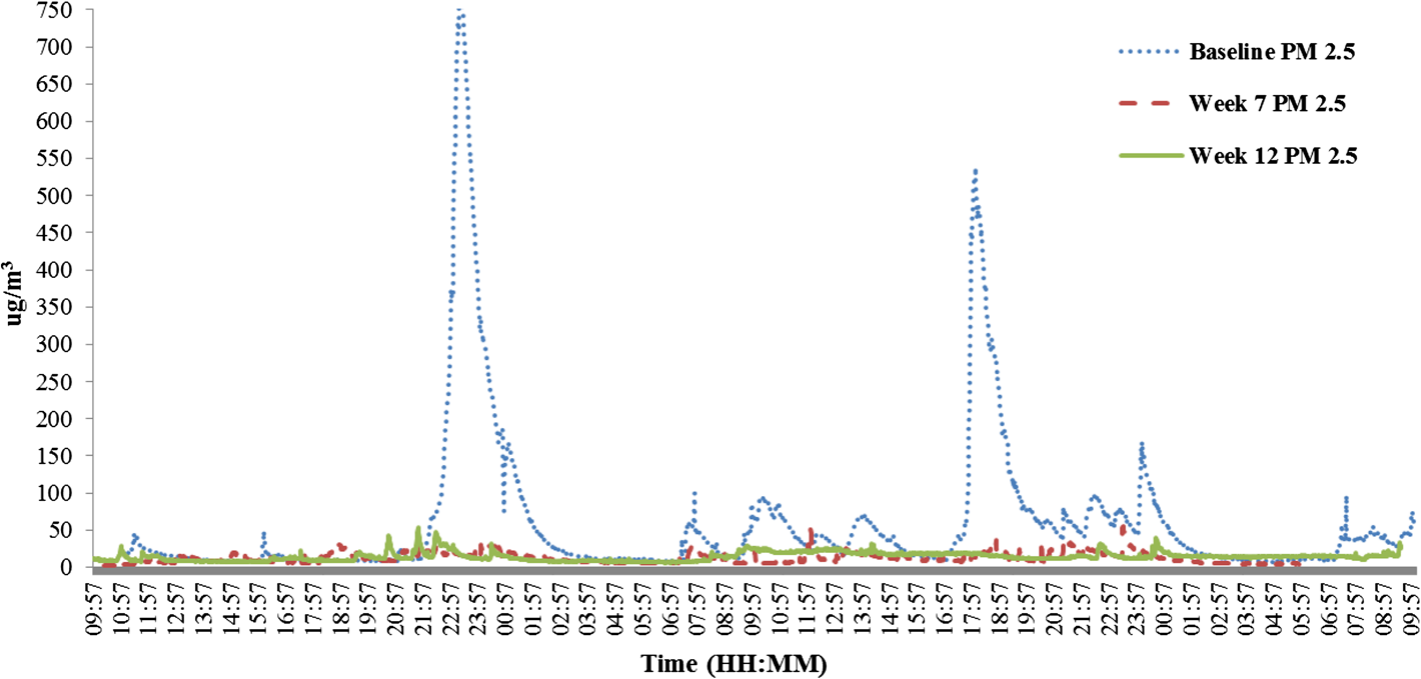
An example of a home air quality (PM_2.5_) feedback graph

Urine sample collection was removed from the protocol for phase 2 given the lack of acceptability and feasibility of this as an outcome measure. Although saliva sample collection was feasible and acceptable, the variability in results led the research team to explore alternative personalised feedback options for phase 2. Saliva samples were still collected at four time points (baseline and weeks 3, 7 and 12), but the results were not fed back to participants unless requested at the end of the intervention period (12 weeks). Building on the findings from the REFRESH study [14] which showed that it was feasible and acceptable to use home air quality data (PM_2.5_) in SFH interventions, personalised feedback was given to participants using PM_2.5_. Home air quality data were collected at three time points (weeks 1, 7 and 12). Data were collected for 48 h prior to the intervention visit, and findings were uploaded immediately (with the exception of week 1 when data were collected at the previous baseline visit) at the end of this collection period to a laptop during the home visit and personalised graphical feedback on home air quality provided to caregivers using Microsoft Excel (see Fig. 6 for an example) during the visit.

## Methods

### Phase 2 procedure

#### Design, setting and recruitment

Phase 2 ran between March and August 2012. The participants were again recruited via CCs, and the inclusion criteria remained the same other than we included caregivers who stated that they were interested in quitting smoking to maximise potential recruitment.

### Phase 2 intervention (see Fig. 1)

Families received a 12-week home-based intervention which aimed to support caregivers to reduce their children’s exposure to SHS in the home and comprised of:

- Four sessions of face-to-face behavioural support (up to 60 min each) and two proactive telephone behaviour support sessions (up to 20 min each) to promote changes to home smoking behaviours based on the principles of motivational interviewing, delivered by a specialist SFH advisor. The support included caregiver education on the dangers of SHS, discussion of smoking behaviour and smoking rules in households and identifying barriers to change and working with participants to elicit strategies to overcome barriers.
- Up to an 11-week supply of NRT to support temporary abstinence in the home (rather than cessation) by caregivers/other household adults. The participants were able to sample all available products during visit two and via discussion with the SFH advisor made an informed choice about product/s. All were supplied at no cost to the participants.
- Graphical and verbal feedback on home air quality (PM_2.5_) at three time points (weeks 1, 7 and 12).

### Data collection methods and analysis

#### Quantitative data

##### Demographic questionnaires

The same questionnaire data were collected as in phase 1, although questionnaires were administered at slightly different time points (baseline and weeks 3, 7 and 12).

##### Cotinine measurement and feedback

All 18 saliva samples were successfully collected and analysed. The average saliva cotinine for the six participants at each time point is shown in Fig. 4. On average, saliva cotinine increased by 1.3 ng/ml (range −16.0 to 17.5 ng/ml) between baseline and week 12. The same measurement procedures to collect saliva samples as in phase 1 were used. Feedback, in the same format as that in phase 1, was only given to caregivers if requested at the end of the intervention period (12 weeks).

##### Air quality measurement and feedback

The concentration of airborne particulate matter <2.5 μm (PM_2.5_) was measured in the main living area of each household using a SidePak AM510 Personal Aerosol Monitor for up to 48 h at baseline, week 7, and week 12 (feedback was given at weeks 1, 7, and 12, see Fig. 6 for an example). Analysis was based on 24 h of sampling in order to compare results to the World Health Organisation’s (WHO) indoor air quality guidelines [23]. The first and last 10 min of the 24-h sampling time were disregarded to reduce potential bias, as a researcher was often present during these periods. Maximum, minimum, and average levels of PM2.5 were calculated, having applied a calibration factor of 0.3 to the raw data (to account for the lower density of SHS aerosol compared to the road test dust used to calibrate the device), and the results were compared to previous levels for week 7 and 12 visits. The amount of time that PM_2.5_ levels exceeded the WHO 24 h guideline of 25 μm/m^3^ [23] was also reported.

##### Quantitative analysis

Data from the interviewer administered questionnaires were captured using SPSS version 14 and descriptive statistics calculated. Raw cotinine data were inputted into Microsoft Excel and descriptive statistics calculated. SidePak PM_2.5_ data were analysed in Microsoft Excel and data were log transformed to normalised distributions. The percentage changes in average (μm/m^3^), maximum (μm/m^3^), and time (minutes) over 25 μm/m^3^ (WHO indoor 24-h guideline) between baseline and week 12 in air quality were calculated.

#### Qualitative data

##### Evaluation interviews

Interviews for phase 2 followed the same procedures as for phase 1 with only a small change to the interview guide to explore the feasibility and acceptability of home air quality measurements and feedback.

##### Qualitative analysis

The same methods as phase 1 were used to thematically analyse the interview transcripts from phase 2.

## Results phase 2

### Recruitment and retention

The second phase of recruitment took 9 weeks and 26 CC sessions; 197 people were approached to assess eligibility and 11 (6 %) met the inclusion criteria, of whom six (55 %) were recruited with none lost to follow-up. Highlighting an improvement in the ability to recruit and retain participants between phase 1 and phase 2. Only a small number of those caregivers who were approached to participate in the study were eligible as the majority reported being a non-smoker and of those who reported smoking, only a small number reported smoking inside the house. The overall retention rate across the two phases was 86 %. No additional smoking adults living in the study households were recruited in phase 2.

Semi-structured evaluation interviews were undertaken with four of the six participants. The participants who did not complete an evaluation interview were either not available to participate within the 2-week window at the end of the intervention or declined to take part.

### Study population

The participant baseline characteristics are presented in Table 2. Phase 1 and 2 participants were similar in age, education, housing and employment status but showed a difference in the number of cigarettes smoked per day.

#### Acceptability and feasibility of behavioural support

Behavioural support discussions lasted between 20 and 60 min and were built around personalised feedback in the form of PM2.5. At 12 weeks, all caregivers self-reported that the behavioural support component was very (*n* = 4) or extremely (*n* = 2) helpful in supporting them to make their home smoke-free. The six participants ranked the behavioural support as the most (*n* = 1) and the second (*n* = 3) or the third (*n* = 2) most effective and important component of the intervention package. Compared to phase 1, behavioural support appeared to be viewed as a slightly less important component of the overall intervention, although in the interviews the participants described similar, positive experiences aligned with the results from phase 1. The participants were also positive about the inclusion of additional, proactive telephone support, which provided further support around creating and maintaining a SFH aligned with behavioural support delivered during the face-to-face sessions and was important during the early stages and served to reassure them that support was available and that they were not alone (Fig. 2c).

#### Acceptability and feasibility of nicotine replacement therapy

At baseline, three caregivers said that they planned to take up the offer of NRT, two were unsure and one was contraindicated (due to breast feeding) and so was unable to accept the offer. Between baseline and week 3, five participants had taken up the offer of NRT and reported using the product/s (two were using quick mist and lozenges; two were using a combination of inhalator, lozenges and quick mist; and one was using the inhalator and quick mist). Between weeks 3 and 7, five participants reported that they had taken up the offer of NRT and reported using the product/s (one was using lozenges, one was using gum, one was using lozenges and quick mist, one was using quick mist, and one was using an inhalator and lozenges). Between weeks 7 and 12, five participants took up the offer of NRT and all five reported using the product/s (one was using lozenges; one was using gum; one was using a combination of an inhalator, lozenges and quick mist; one was using quick mist; and one was using an inhalator and lozenges). The participants reported predominantly using NRT to help with cravings for short period of time and for helping them to cut down without planning to quit smoking. Views on NRT were very similar to that of the participants in phase 1 with a number of participants reporting more significant side effects (Fig. 3c).

#### Acceptability and feasibility of personalised feedback

##### Cotinine

All 18 saliva samples were successfully collected and analysed. On average, saliva cotinine increased by 1.3 ng/ml (range −16.0 to 17.5 ng/ml) between baseline and week 12. The participants reported, as part of the interviews, that collecting saliva from children was acceptable and non-intrusive in line with phase 1.

##### Home air quality

Twenty-four-hour log-transformed data showed that compared to baseline, week 12 readings were on average 49 % less in average levels of PM_2.5_, 74 % less in the maximum level recorded and 75 % less in the total time PM_2.5_ levels were over the WHO 24 h recommended level of 25 μg/m^3^ [23]. At week 12, four out of six participants ranked the personalised feedback as the most effective and important component of the intervention. Monitoring of home air quality was generally feasible and accepted, with only a minority of participants expressing concerns about the noise made by the monitor. The participants felt that they were able to understand the results and they appreciated the personal, visual and ‘real-time’ nature of the graphical and numerical feedback. Similar to the saliva cotinine feedback, at first, the air monitor results caused some participants to express ‘shock’ at the levels of PM_2.5_ detected in their home (Fig. 5c). The participants remarked that smoking in a different room to where the air quality monitor was positioned still had a negative impact on the air quality readings and that it often took several hours after smoking for the PM_2.5_ levels to fall below the WHO 24 h safe average of 25 μm/m^3^ [23]. As a result, the caregivers were motivated to create a SFH and reduce the levels of PM_2.5_ that the air quality monitor detected in the home throughout the study (Fig. 5d).

## Discussion

This study has demonstrated that this novel intervention was feasible and acceptable to disadvantaged families to help them to reduce their children’s exposure to SHS at home.

Qualitative findings highlighted that personalised feedback of children’s levels of SHS appeared to motivate behavioural change and that home air quality feedback was the more reliable and acceptable method for doing this.

All participants appreciated the behavioural support component of the intervention with some showing particular appreciation for the non-judgemental manner in which it was delivered. The literature suggests that increasing the intensity of home-based interventions can increase their effectiveness [13]. The intervention was made more intensive in phase 2 by adding proactive telephone support and increasing the frequency of home visits in the earlier stages of the intervention. It is possible that the intensive nature of the intervention and the regular, personalised behavioural support encouraged strong relationships to be built and therefore contributed to the good retention of study participants. The changes to the structure of the intervention for phase 2 were also made to try and aid the transition to independence for participants by making visits less frequent towards the end of the intervention attempting to encourage participants to maintain changes on their own.

All participants not contraindicated accepted the initial offer of NRT for temporary abstinence but some expressed negative views mainly around the side effects, such as taste, which have been reported in other studies [16]. However, other participants suggested that they may go on to use NRT for a quit attempt in the future, possibly as a result of their positive experience of using NRT to temporarily abstain from smoking in the home and so it is important that NRT is included as part of the intervention in an exploratory efficacy trial.

Providing feedback via children’s cotinine whilst acceptable and feasible (for saliva) proved to be problematic. Results were variable, often not consistent with corresponding urine cotinine results, and did not reflect the home smoking behaviour changes self-reported by the participants. Ultimately, this variability had a detrimental effect on caregivers’ motivations to maintain any changes to home smoking behaviours. A further limitation of cotinine feedback is that it does not relate exclusively to SHS exposure in the index household as levels can be impacted by SHS exposures from other environments (such as if the child spends considerable time in a grandparent’s home where smoking was unrestricted). This issue limits the effectiveness of saliva cotinine feedback as an outcome measure for SFH interventions and as a motivator for sustainable behaviour change.

In an attempt to overcome the issues with cotinine, home air quality data (PM_2.5_) were used to provide personalised feedback in phase 2. The graphical presentation of results was well received and understood, supporting findings from previous research which shows that smoking caregivers are able to understand complex data presented to them in this manner and that this type of feedback is acceptable and motivating to caregivers [14]. It appears that the use of longitudinal air quality data helped to motivate caregivers to initiate and maintain changes to their home smoking behaviours. By providing feedback on three occasions over the 12-week intervention period, the participants were able to see the positive effects that their behavioural changes had on home air quality. There were however some potential issues with using home air quality, for example, monitors were placed in one specific location within a household and we were therefore unable to accurately indicate air quality levels in other areas of the house, although this location was identified as the place where the child spent the most amount of time during the day. It is also possible that participating families could change their smoking behaviour inside the house for the period of time that the monitor was present [14] although the participants reported that they often forgot that the monitor was there and so it is unlikely that this would have significantly influence the results. Finally, it is important to highlight that whilst we have used the term SHS exposure throughout this manuscript, PM_2.5_ is a proxy measure of personal exposure and an indirect measure of SHS that is not specific to cigarette smoking as results can be influenced by any airborne particles <2.5 μm in size.

The findings from the current study help to build on the results of the REFRESH intervention feasibility study [14, 24], although REFRESH did not offer caregivers NRT, only provided home air quality feedback on two occasions (compared to three in the current study) and the intervention period was significantly shorter (4 vs. 12 weeks). The REFRESH results showed a statistically significant decrease in maximum PM_2.5_ between weeks 2 and 4 within the intervention group but no significant difference between the intervention and comparator arm [14]. Since the evidence base suggests that intensive and sustained contact is more likely to be successful in helping smoking caregivers to make changes [13], we might expect our intervention to be more effective. This is supported by the feasibility data which showed an average decrease of 49 % between baseline and week 12 in average PM_2.5_ and a 74 % reduction in the maximum PM_2.5_.

Recruitment proved labour intensive with a total of 65 Sure Start Children’s Centre sessions being attended in order to recruit 14 participants. Only a small minority (7 %) of the caregivers approached met the inclusion criteria during the initial recruitment discussion about the study. This is lower than we had anticipated given the relatively high smoking rates of caregivers accessing CCs [17] and that 39 % of children living with smokers in England are reportedly exposed to SHS at home [6]. However, it may reflect the nature of recruitment in CCs (sometimes in a public environment where the discussion could be overheard) and the potential stigma attached to smoking in the home with young children. The caregivers may therefore have been smoking at home but not willing to share this information with the CC staff and/or research team and thus would be deemed ineligible. In addition, we know that rules about smoking at home are often fluid and that whilst caregivers may initially state that they always smoke outside, it is only with further exploration of home smoking rules that it becomes apparent that caregivers sometimes smoke inside [17]. To try and overcome this in the explanatory RCT, a number of additional recruitment strategies will be employed (including, for example, via health visitor mail shots, outreach services, advertising, the local stop smoking SFHs service) and more care taken to ensure that recruitment discussions are conducted more privately where possible. Having said this, nearly half of eligible participants took part in the study and 12 of the 14 participating families completed the intervention. This suggests that a proportion of smoking caregivers are interested in making changes to their home smoking behaviours and that the home setting, length and delivery of the intervention were acceptable to them.

## Conclusions

Accessing disadvantaged smoking caregivers was labour intensive but once recruited, the caregivers found this novel intervention both feasible and acceptable in supporting them to protect their children from SHS exposure at home. Non-judgemental support and encouragement over time, supplemented with personalised home air quality feedback, are key to motivating caregivers to initiate and maintain changes to their home smoking behaviours. The intervention tested in phase 2 of this feasibility study did not require further changes following detailed evaluation and is now being tested for efficacy and cost-effectiveness in an exploratory RCT.

## Additional file

Additional file 1: Caregiver week 12 interview guide.

## Abbreviations

CCs: Children’s centres
NRT: Nicotine replacement therapy
PM_2.5_: Particulate matter <2.5 μm
SHS: Secondhand smoke
SFH: Smoke-free home
