# Supplementary material for: Protecting children from secondhand smoke: a mixed-methods feasibility study of a novel smoke-free home intervention
Source: Pilot Feasibility Stud. 2016 Sep 12;2:53. doi: 10.1186/s40814-016-0094-7 (PMC5153871; doi:10.1186/s40814-016-0094-7)
Supplement: Additional file 1: — Caregiver week 12 interview guide. [file 40814_2016_94_MOESM1_ESM.pdf]

University of Nottingham
School of Community Health Sciences
Division of Epidemiology and Public Health
Clinical Sciences Building
Nottingham City Hospital
Nottingham
NG5 1PB

Title of Project: A study to test the feasibility of an intervention to reduce children's exposure to second hand smoke in the home

Name of Investigators: Professor Sarah Lewis, Dr Laura Jones and Miss Sarah Field-Richards

Caregiver Week 12 Interview Guide

Introduction

- Thank for continuing to take part in the study.
- Check consent form has been signed. Keep a copy and give a copy back to the caregiver.
- Statement on confidentiality, right to withdraw consent, recording of the interview.
- Review the purpose of the study in general:
    - To find out more about homes where children may be exposed to second hand smoking; caregiver's perspective on smoking
    - Interested in your views and experiences of using the Smoke-free Homes support package over the last 12 weeks
    - The information will be used to modify and refine the package so that it is as effective as possible in helping other smokers (like yourself) to reduce children's exposure to second hand smoke.
    - Emphasize the value of their views and opinions in helping us to develop the most effective package.
- Ask if the participants have any questions before starting the interview.

General

1. So we've come to the end of the 12 weeks of the study. How has the experience of creating a smoke-free home been?
2. Is there anything that you've found difficult? (if yes, tell me about that)
3. Is there anything that you feel has helped you? (if yes, tell me about that)
4. Is there anything else that you feel we could have provided which would have helped you create a smoke-free home?

Questionnaires

Over the last 12 weeks, you've completed four questionnaires where the questions were read out to you by either one of the researchers or the smoke-free homes advisors.

1. Is there anything you'd like to comment on to do with the questionnaires?
2. What are your overall feelings about the questionnaires?
3. Can you suggest anything that we could do to improve the questionnaires?

Behavioural support

1. Overall, do you feel that the behavioural support provided by the smoke-free homes advisors has been useful in helping you create a smoke-free home?
   If yes,
   i) Can you explain why/how you found it useful?

   If no,
   ii) Can you explain why you think that you didn't find it useful?
2. Could you suggest any ways in which we improve the behavioural support element of the package?
3. Have you used the additional text or telephone service provided by the smoke-free home advisors?
   If yes,
   i) How often have you used these services?
   ii) Have you found them helpful?
   iii) Is there anything that could improve this service?

   If no,
   i) Are there any specific reasons why you chose not to use the service?
   ii) Is there anything that could be changed that would make it more likely that you would use the service?

NRT

1. Can you tell me a bit about your use of NRT during the last 12 weeks?
2. Have you found using NRT useful in helping you to create a smoke-free home?
3. Have you had enough NRT to last you between the smoke-free advisors visits? (if they ran out, ask what action they took)
4. Is there anything surrounding the NRT component of the package that you think we could change to make it more effective in helping people to create and maintain a smoke-free home.

Biochemical feedback

1. How have you felt about receiving the feedback from your child's urine and saliva samples?
2. How do you feel about the biochemical feedback element of the package overall?
3. Is there anything that you would change to improve the way the samples are collected or the way in which the feedback it is provided?

4. Were you able to discuss the feedback with your behavioural support advisor? Was this useful?
5. Do you feel that the biochemical feedback that you have received has helped you in creating a smoke-free home?

Education and information

Diary

1. What are your overall thoughts about using the diary over the last 12 weeks?
2. Are there any pages in the booklet that you have used more than others?
3. How helpful have you found it to be in supporting you to maintain a smoke-free home?
4. Do you have any suggestions as to how we can improve the pack?

DVD

1. Have you watched the DVD?

If yes,

- i) Can you tell me about your overall thoughts on them?
- ii) Is there anything that you thought was particularly good or bad about then?
- iii) Can you offer any suggestions as to how they could be improved?
- iv) Have you used the resource pack that accompanied the DVD? (if yes, in what way have they used it?)
- v) Have you watched the DVD more than once in the last 12 weeks?

If no,

- i) Are there any specific reasons why you chose not to watch the DVD/ Is there anything that put you off watching the DVD?

General

1. In your opinion, has participation in the study matched what you were told participation would involve at the start of the study? If no, how has it been different?
2. If you had the chance to participate in the study again, would you participate?

Conclusion

- Tell the participants that they have reached the end of the interview
- Do they have any questions in return?
- Remind them about confidentiality.
- Thank them for their time.
- Give people inconvenience allowance (retail vouchers) and get signatures when these are handed over.
